# *S*-maup: Statistical test to measure the sensitivity to the modifiable areal unit problem

**DOI:** 10.1371/journal.pone.0207377

**Published:** 2018-11-27

**Authors:** Juan C. Duque, Henry Laniado, Adriano Polo

**Affiliations:** 1 Department of Mathematical Sciences, Universidad EAFIT, Medellin, Colombia; 2 RiSE-group, Universidad EAFIT, Medellin, Colombia; 3 Department of Economics, Universidad EAFIT, Medellin, Colombia; Stony Brook University, Graduate Program in Public Health, UNITED STATES

## Abstract

This work presents a nonparametric statistical test, *S*-maup, to measure the sensitivity of a spatially intensive variable to the effects of the Modifiable Areal Unit Problem (MAUP). To the best of our knowledge, *S*-maup is the first statistic of its type and focuses on determining how much the distribution of the variable, at its highest level of spatial disaggregation, will change when it is spatially aggregated. Through a computational experiment, we obtain the basis for the design of the statistical test under the null hypothesis of non-sensitivity to MAUP. We performed an exhaustive simulation study for approaching the empirical distribution of the statistical test, obtaining its critical values, and computing its power and size. The results indicate that, in general, both the statistical size and power improve with increasing sample size. Finally, for illustrative purposes, an empirical application is made using the Mincer equation in South Africa, where starting from 206 municipalities, the *S*-maup statistic is used to find the maximum level of spatial aggregation that avoids the negative consequences of the MAUP.

## Introduction

Although spatial data are increasingly disaggregated, many socioeconomic studies require some level of aggregation (e.g., neighborhoods, municipalities, states, districts, countries). Spatial aggregation is useful for calculating rates and indexes, minimizing the influence of outliers, or preserving confidentiality [1, 2]. Spatial aggregation is also useful for creating meaningful units for analysis [3, 4], reducing computational complexity [5], controlling for spurious spatial autocorrelation [6, 7], comparing results at different scales [8, 9], and merging different datasets to a comparable resolution [10].

However, spatial aggregation triggers a problem known as the Modifiable Areal Unit Problem (MAUP). The MAUP, introduced in the literature by [11] and [12], refers to the sensitivity of statistical results to changes in the spatial units of analysis. The MAUP has two dimensions: the scale effect and the zoning effect. The scale effect refers to changes in the size of the spatial units, which implies a change in the number of spatial units, e.g., doing the analysis at the state or county level. The zoning effect refers to changes in the shape of the spatial units preserving the number of units, e.g., aggregating USA counties into 50 states is merely one of the many ways in which one can aggregate counties into 50 spatial units.

Although the literature on MAUP is extensive, to the best of our knowledge, there is no statistical tool that allows a practitioner to easily determine the level of sensitivity of a spatially intensive variable to the MAUP (in Geographic Information Science, spatially intensive variables, such as rates, densities and proportions, are averaged when areas are aggregated [13]). Hence, in this paper, we present *S*-maup, a nonparametric statistical test to measure the sensitivity of a spatially intensive variable to the MAUP. Instead of looking at a specific measure of central tendency or dispersion or at the coefficient associated with the variable in a specific regression, *S*-maup focuses on determining how much the distribution of the variable, at its highest level of spatial disaggregation, will change when it is aggregated into a given number of regions. For its calculation *S*-maup requires the number of areas, the *ρ* parameter, that measures the degree of spatial correlation of the variable, and the number of regions in which the areas will be aggregated. Under the null hypothesis of non-sensitivity to MAUP, *S*-maup would be useful for determining the maximum level of aggregation that we can apply to a given variable before it loses its distributional characteristics. *S*-maup could also be used to determine whether the results obtained at a given scale (e.g., counties) hold for another scale (e.g., states).

The rest of this article is structured as follows. We begin with a literature review concerning the primary research surrounding the MAUP. We then explore the effects of the MAUP through a computational experiment. Next, we propose a test statistic, *S*-maup, and its empirical distribution under the null hypothesis of non-sensitivity to MAUP. Next, we establish the power and size of the statistic under various levels of spatial autocorrelation and number of areas. We then present a simple example of the use of the *S*-maup statistic. Last, we conclude and suggest avenues for further investigation.

## Literature review

The effects of aggregating spatial data have been a subject of study since the early 1930s and have been referred to by different names, such as aggregation effects [14], scale problem [3], ecological fallacy [15], and Modifiable Areal Unit Problem, MAUP, [12]. If one delves into the details, it can be argued that these previous concepts are different. However, these concepts possess as a common factor a concern regarding the undesired effects that result from working with aggregate data. Hereinafter, we will refer to this problem as MAUP.

The literature on MAUP can be divided into three blocks: first, definition of the problem [12, 16, 17]; second, measurement of its effects on statistics such as the mean [18, 19], median and standard deviation [6], variance and covariance [20, 21], and correlation coefficient [3, 12, 14, 22]; and last, potential ways to minimize the aggregation effects [17, 23–26].

It is well known that the impact of the MAUP on the mean can be considered negligible [17–20]. However, the MAUP has a large impact on the variance, which decreases when the variable exhibit high values of spatial autocorrelation [21]. With respect to the statistical association, such as the covariance and correlation coefficient, [22], [12] and [17] found that the sensitivity to MAUP increases as the level of spatial aggregation increases (scale effect), i.e., the correlation between variables *X* and *Y* will exhibit a wider variation if, for example, USA counties are aggregated into 50 spatial units than if they were aggregated into 1,000 spatial units.

The MAUP effects have also been studied in OLS regressions [9, 11, 22, 27], logit models [28], Poisson regression [29], spatial interaction models [30], spatial econometrics models [31], forecasts in regional economy [32], and spatial autocorrelation statistics, such as the Moran’s coefficient, Geary’s Ratio, and G-Statistic [28, 33, 34]. Other authors have studied the MAUP effects in more sophisticated methods, such as the factorial analysis [35], spatial interpolation [36], image classification [37], location and allocation models [38], and discrete selection models [39].

Although there is no solution to the MAUP because it is inherent to the use of spatial data, some authors have proposed different alternatives to minimize its effects: the formulation of scale-robust statistics [40], the design of optimal aggregations that minimize the loss of information [4, 9, 16, 41, 42], the use of a set of auxiliary or grouping variables together with variables at the individual level [43, 44], and the measurement of rates of change through the concept of a fractal dimension [24].

Most studies above required extensive computational experiments. Table 1 summarizes the main characteristics of those experiments, including the covered dimensions (scale or zoning), studied statistics (mean, variance, correlation, regression coefficients, etc.), type of data (real or simulated), studied variables (income, rates, random, etc.), and size of the experiment in terms of the number of areas and regions (herein, we will refer to area as the smallest spatial unit of observation and region as the spatial units that result from aggregating the areas into contiguous spatial units). From this table, we can highlight the dominance of the use of real data over simulated data and the evident increase in the size of the experiment as the computational capacity increases over the years. As expected, the two driver parameters in these experiments are the number of areas and the number of regions. Although it has been considered in a few experiment [6, 12, 21], the level of spatial autocorrelation of the variables/attributes being aggregated plays an important role in the level of sensitivity of the variable to the MAUP. Finally, the mean is significantly highlighted by being the more common grouping operator, i.e., if areas *i* and *j*, with attribute values *X*_*i*_ and *X*_*j*_, are merged into a region, the attribute value for the resulting region is calculated as the mean of *X*_*i*_ and *X*_*j*_, which indicates that all of the experiments use spatially intensive variables.

**Table 1:** Computational experiments on MAUP.

Author (Year)	Dimension / Effect on…	Grouping operator	Data	Variable	Size
[14]	Scale / *r*_*xy*_	Sum	Census Tracts in Cleveland	Male juvenile delinquency and monthly income. Agricultural products and the number of farmers	1) 252 areas into 200, 175, 150, 125, 100, 50, and 25 regions 2) 1,000 areas into 63, 40, 31, and 8 regions
[15]	Scale / *r*_*xy*_	Proportions	Nine geographic divisions of the USA in 1930	Race and illiteracy	97,272 individuals into 9 regions
[3]	Scale / *r*_*xy*_	Mean	Agricultural counties in England	Production of wheat and potatoes per acre	48 areas into 24, 12, 6, and 3 regions
[22]	Scale / *r*_*xy*_	Mean	Metropolitan area of Los Angeles	Household income and education level of the head of household	1,556 census tracts into 134 Welfare Planning Council Study areas and 35 Regional Planning Commission Statistical Areas
[12]	Scale—Zoning / *r*_*xy*_	-	Counties in Iowa and simulated data with *ρ*_+_, *ρ*_0_, and *ρ*_−_	% of Republican votes and % population over 60 years.	99 areas into 6, 12, 18, 24, 30, 36, 42, 48, 54, 60, 66, and 72 regions
[17]	Scale—Zoning / *μ*, *σ*^2^, *σ*, *ρ*	Mean	Quadrat in Hukuno Town, Japan and weights of wheat plots of grain	Quadrat counts of houses and weights of wheat plots of grain	1) Regular lattice of 32x32 into 16x16, 8x8, 4x4, and 2x2 regions 2) Regular lattice of 25x20 cells into 8x8, 4x4, and 2x2 regions
[28]	Scale—Zoning / *β*′*s*	Mean and Proportion	Metropolitan area of Buffalo	Household income, % of population per area, % of population over 65 years	871 areas into 800, 400, 200, 100, 50, and 25 regions
[18, 19]	Zoning / *μ*, *σ*^2^, *r*_*xy*_ y *β*	Mean and weighted average	Regular lattices	Simulated data with Uniform, Normal and Poisson distribution	10,000 areas into 10x10, 7x7, and 3x3 regions
[8]	Zoning / *σ*^2^	Mean	City of Adelaide, Australia	82 socioeconomic variables	917,000 people into 1,584 districts
[20]	Zoning / *σ*^2^	Mean	Lancashire, UK	8 census variables	304 areas into 137, 122, 106, 91, 76, 61, 46, and 30 regions
[27]	Scale—Zoning / *r*_*xy*_, *β*	Mean	UK	Census variables and simulated variables	Regular lattice of 120x120 into 1x1, 2x2, 3x3, 4x4, and 5x5 regions
[33]	Scale / *I* − *Moran* and *G* − *Statistic*	Mean	Malasia	Biomass areas and elevation data	Regular Lattice of 220x188 into 2x2, 3x3, 4x4, …, and 20x20 regions
[34]	Scale / *I* − *Moran* and *G* − *Statistic*	Mean	Manitoba, Canada	Normalized Difference Vegetation Index (NDVI)	Regular lattice of 300x300 into 3x3, 5x5, 7x7, 9x9, 11x11, 13x13, and 15x15 areas
[21]	Zoning / *σ*^2^, *r*_*xy*_, and *β*	Mean	Regular lattices	Simulated variables with different levels of spatial autocorrelation and variance	400 areas into 180, 160, 140, 120, 100, 80, 60, and 40 regions
[6]	Zoning / *σ*	Mean and Median	Regular lattices	Simulated data with different levels of spatial autocorrelation	Regular lattice of 512x512 into 3x3, 9x9, 11x11, 21x21, 31x31, 41x41, 51x51, 61x61, 71x71, and 81x81 pixel window sizes
[30, 31]	Scale / *β*	Mean	Regular lattice	Simulated data	Regular lattice of 64x64 into 32x32, 16x16, 8x8, and 4x4.

*r*_*xy*_: Correlation, *μ*: Media, *σ*^2^: Variance, *σ*: Covariance, *ρ*: Spatial autocorrelation, *β*: Regression coefficients.

Based on the available literature, a practitioner can anticipate high(low) variation of its results when the aggregation level is high(low) and the level of spatial autocorrelation of its variable is low(high). However, there is no tool in the literature that allows the assignment of a specific number and statistical significance to that variation. The closest the research can get to that number would require a computational experiment involving the calculation of the results for a large number of random aggregations of the areas into a predefined number of regions. This paper constitutes the very first attempt to formulate a nonparametric statistical test to easily measure the sensitivity of a spatially intensive variable to the MAUP.

## MAUP effects

In this section, we design a computational experiment to identify the key elements that should be included in the construction of the statistical test. Following previous experiments in the literature on the MAUP effects (e.g., [20] and [31]), we consider the two main parameters involved in the exploration of scale and zoning effects: number of areas (*N*) and number of regions (*k*). As in [12] and [21], we also take into account different levels of spatial autocorrelation, *ρ*.

**Fig 1:**
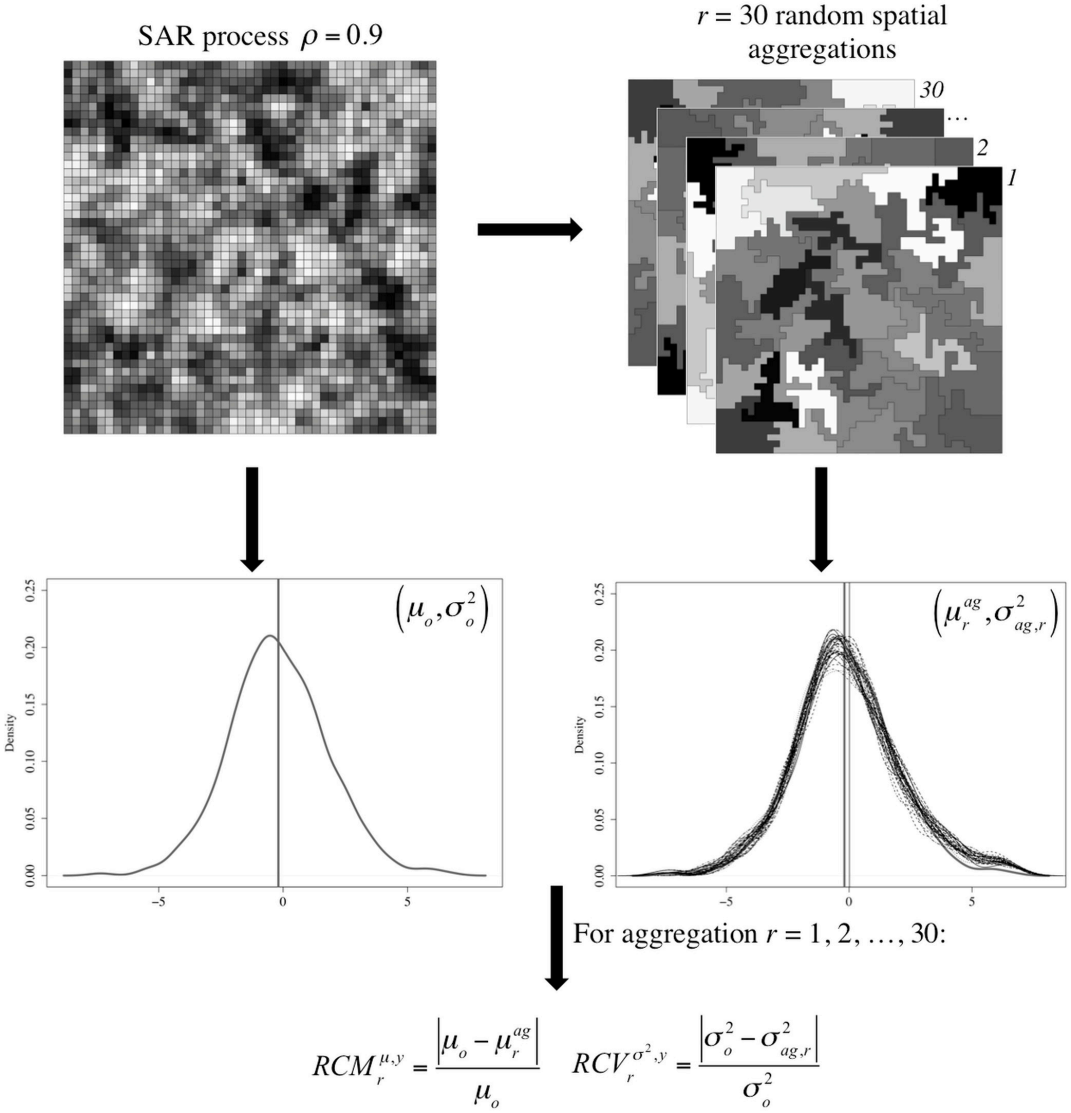
Instance of the experiment.

Fig 1 summarizes the steps followed to generate an instance of the computational experiment: (1) *y*^*ρ* = 0.9^ is a random variable generated by a Spatial Autoregressive (SAR) process with autoregressive parameter *ρ* = 0.9 and rook contiguity matrix. (2) The areas are randomly aggregated into *k* spatially contiguous regions using a seed-based region growing algorithm proposed by [45]. The attribute value for each region is calculated as the mean value of the attribute values of the areas assigned to the region. This random aggregation is repeated *r* = 30 times, so that we generate 30 different ways to aggregate *N* areas into *k* regions. (3) We calculate the mean and variance of the original, disaggregated, variable as *μ*_*o*_ and $\sigma_{o}^{2}$. (4) We calculate the mean and variance of each one of the aggregated variables as *μ*_*ag*_ and $\sigma_{ag}^{2}$. (5) We calculate the relative change in the mean (*RCM*), Eq (1), and the relative change in the variance (*RCV*), Eq (2), between the original variables and each of the 30 aggregated variables. (6) We summarize the effect of aggregating *N* areas into *k* regions as the mean *RCM*, $\bar{RCM}$, and mean *RCV*, $\bar{RCV}$, using Eqs (3) and (4). We repeat steps (1) to (6) 50 times for each value of *ρ* considered in the experiment.

$$\begin{array}{c} RCM_{r}^{\mu ,y}=\frac{\left|\mu_{o}-\mu_{r}^{ag}\right|}{\mu_{o}} \end{array}$$ (1)

$$\begin{array}{c} RCV_{r}^{\sigma^{2},y}=\frac{\left|\sigma_{o}^{2}-\sigma_{ag,r}^{2}\right|}{\sigma_{o}^{2}} \end{array}$$ (2)

$$\begin{array}{c} \bar{RCM}=\frac{\sum_{r=1}^{30}RCM_{r}^{\mu ,y}}{30} \end{array}$$ (3)

$$\begin{array}{c} \bar{RCV}=\frac{\sum_{r=1}^{30}RCV_{r}^{\sigma^{2},y}}{30} \end{array}$$ (4)

Although there exist many options regarding the definition of neighbors, in this paper we decided to use the rook definition [46], which is the most basic and the most popular specification in the field of regional science [47]. Also, in the field of spatial econometrics, although it is a controversial issue, it is recommended the use of an exogenous weights matrix such as the first-order spatial contiguity matrix [48–50]. The rook matrix has been also used in four of the most important computational experiments on the effects of the MAUP [20, 30, 31, 33]. However, according to [51], in grouping through averages, the variance of the aggregated variable decreases faster as the average number of neighbors increases. Therefore, we highlight that the S-maup is designed for cases in which the average number of neighbors is close to 4 (see [52], for a topological characterization of different specifications of spatial contiguity matrices).

**Fig 2:**
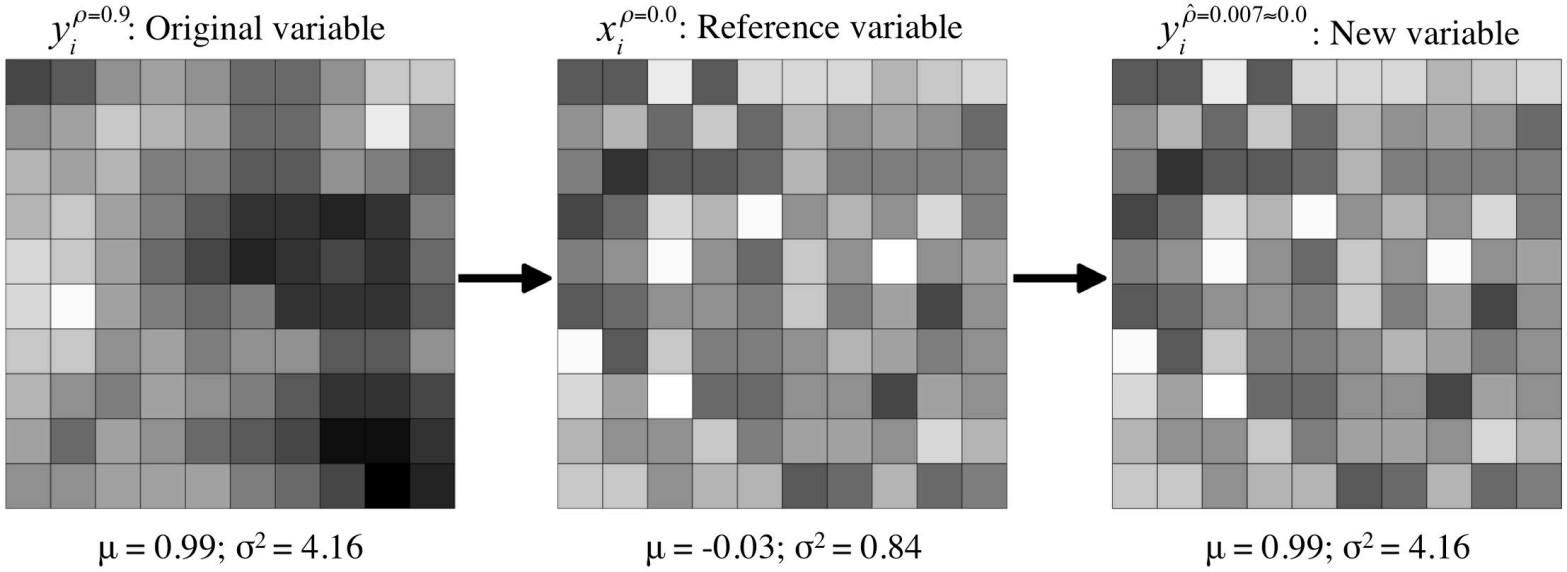
Example of spatial autocorrelation generation.

As is common in the literature on the MAUP effects, our experiment considers different levels of spatial autocorrelation, *ρ*, ranging between -0.9 and 0.9. Each instance of *ρ* = 0.9, *y*^*ρ* = 0.9^, was generated from a spatial autoregressive data generating process, SAR, of the type *y* = *ρWy* + *ϵ*. Once we generate a *y*^*ρ* = 0.9^, we use it to generate instances with other values of *ρ* (e.g., *y*^*ρ* = 0^, *y*^*ρ* = −0.9^, or *y*^*ρ* = 0.5^) by spatially redistributing its values. For example, an instance of *y*^*ρ* = 0.0^ is generated from *y*^*ρ* = 0.9^ according to the following procedure: (1) Generate a SAR process *y*^*ρ* = 0.9^. (2) Generate a SAR process *x*^*ρ* = 0.0^. (3) Perform a spatial redistribution of the values of *y* following the spatial pattern of *x*; i.e., the area with the highest value of *x* takes the highest value of *y*; the area with the second highest value of *x* takes the second highest value of *y*; and so forth. (4) Estimate the *ρ* value from the redistributed variable and keep it if and only if $|\hat{\rho }-\rho |\leq 0.1$; otherwise, repeat steps (2) to (4). With this novel process we guarantee that no aggregation effect attributed to a change in *ρ* comes from using different vectors of values. Fig 2 summarizes the main steps of the process.

Having clarified the process that we follow at each instance and our strategy for generating the *y*^*ρ*^ values, we present the parameters used in the computational experiment:

**Table:** 

*N* =	Number of areas. *N* = {25, 100, 225, 400, 625, 900};
$y_{i}^{\rho }$ =	SAR process with *i* = {1, …, 50}, and *ρ* = {±0.9, ±0.7, ±0.5, ±0.3, 0};
*k*, *K* =	Index and set of number of regions, such that:
	for *N* = 25, *K* = {3, 5, 10, 13, 15, 18, 20, 22, 24}
	for *N* = 100, *K* = {2, 4, 7, 12, 25, 40, 53, 67, 80, 90, 99}
	for *N* = 225, *K* = {3, 5, 10, 15, 30, 60, 90, 120, 150, 180, 200, 220 }
	for *N* = 400, *K* = {4, 9, 18, 26, 50, 110, 160, 213, 267, 320, 360, 396}
	for *N* = 625, *K* = {4, 6, 14, 27, 43, 80, 170, 250, 333, 417, 500, 563, 618}
	for *N* = 900, *K* = {4, 9, 20, 40, 60, 120, 240, 360, 480, 600, 720, 810, 890 };
*r* = 30	Number of random spatial aggregations.

We implemented the experiment in Python [53]. For the spatial aggregations, we use the Python library ClusterPy [54]. We ran the experiment in the supercomputer *APOLO*, at the Center of Scientific Computation (Universidad EAFIT), equipped with a Dell Power Egde 1950 III of 8 cores, 2.33 GHz Intel Xeon that executes Linux Rocks 6.1 to 64 bits.

**Fig 3:**
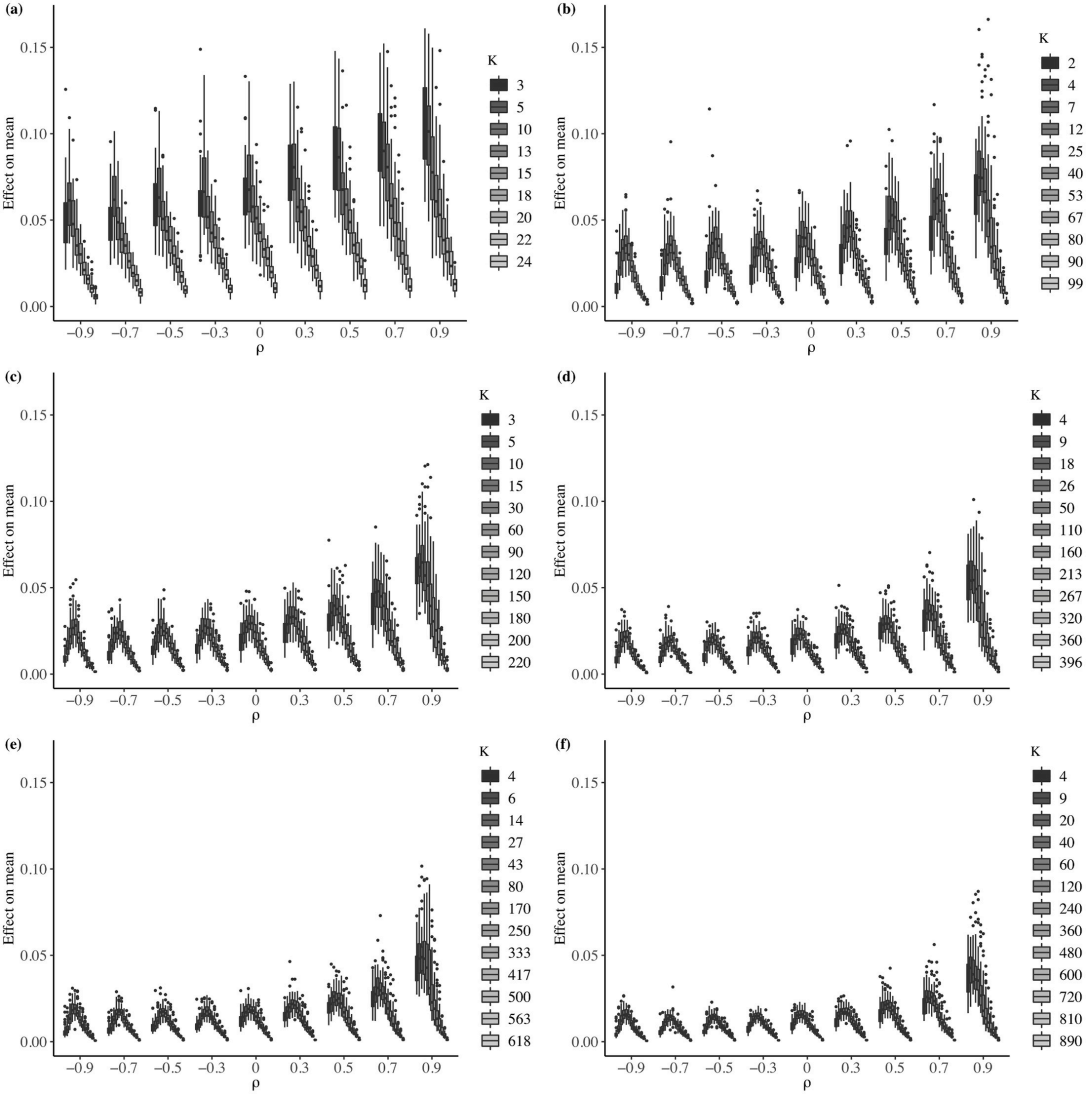
Relative change in mean—Average effect. (a) *N* = 25; (b) *N* = 100; (c) *N* = 225; (d) *N* = 400; (e) *N* = 625; (f) *N* = 900.

Each box plot in Fig 3 summarizes the 50 values of $\bar{RCM}$ calculated for each value of *ρ* and *k*. The maximum bounds value of the vertical axis in the figure show low relative changes in the mean. To make sure that the mean effect can be discarded, we calculate the two-sample *t*-test to compare the mean of each original variable, *μ*_*o*_, with the mean of each aggregated variable, *μ*_*ag*_. We report in Table 2 the proportion of aggregated variables for which the two-sample *t*-test rejected the null hypothesis of *μ*_*o*_ = *μ*_*ag*_ at the 5% level of significance. From this result, we can conclude that there is not a MAUP effect on the mean, which is consistent with those results found by [17, 18] and [20].

**Table 2:** Effect on mean.

	Number of areas					
	*N* = 25	*N* = 100	*N* = 225	*N* = 400	*N* = 625	*N* = 900
Proportion*	0	0.00063	0.00014	0.00041	0.00063	0.0012

*Number of *t*-test rejections divided by |*i*| * |*ρ*| * |*K*| * *r*, where |⋅| indicates the cardinality. It includes aggregations with k ≥ 10.

**Fig 4:**
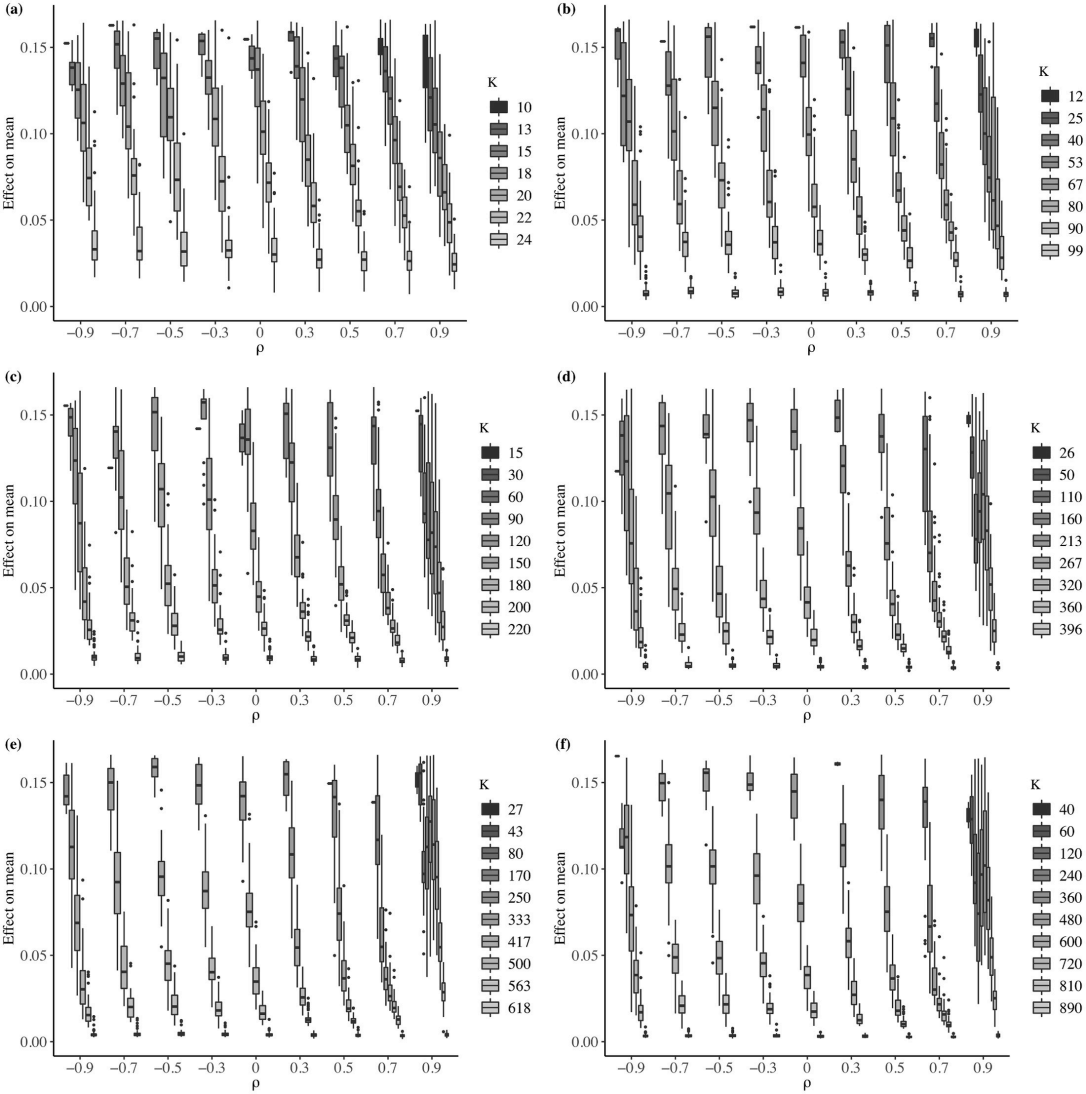
Relative change in variance—Average effect. (a) *N* = 25; (b) *N* = 100; (c) *N* = 225; (d) *N* = 400; (e) *N* = 625; (f) *N* = 900.

Each box plot in Fig 4 summarizes the *i* = 50 values of $\bar{RCV}$ calculated for each value of *ρ* and *k*. Unlike the case seen with the mean, the effect of variance is considerably greater. The box plots show that the effect of MAUP on variance decreases for two reasons: an increase in the level of spatial autocorrelation, *ρ*; and (2) an increase in the number of regions, *k*. These effects on variance are consistent with those found by [21].

**Fig 5:**
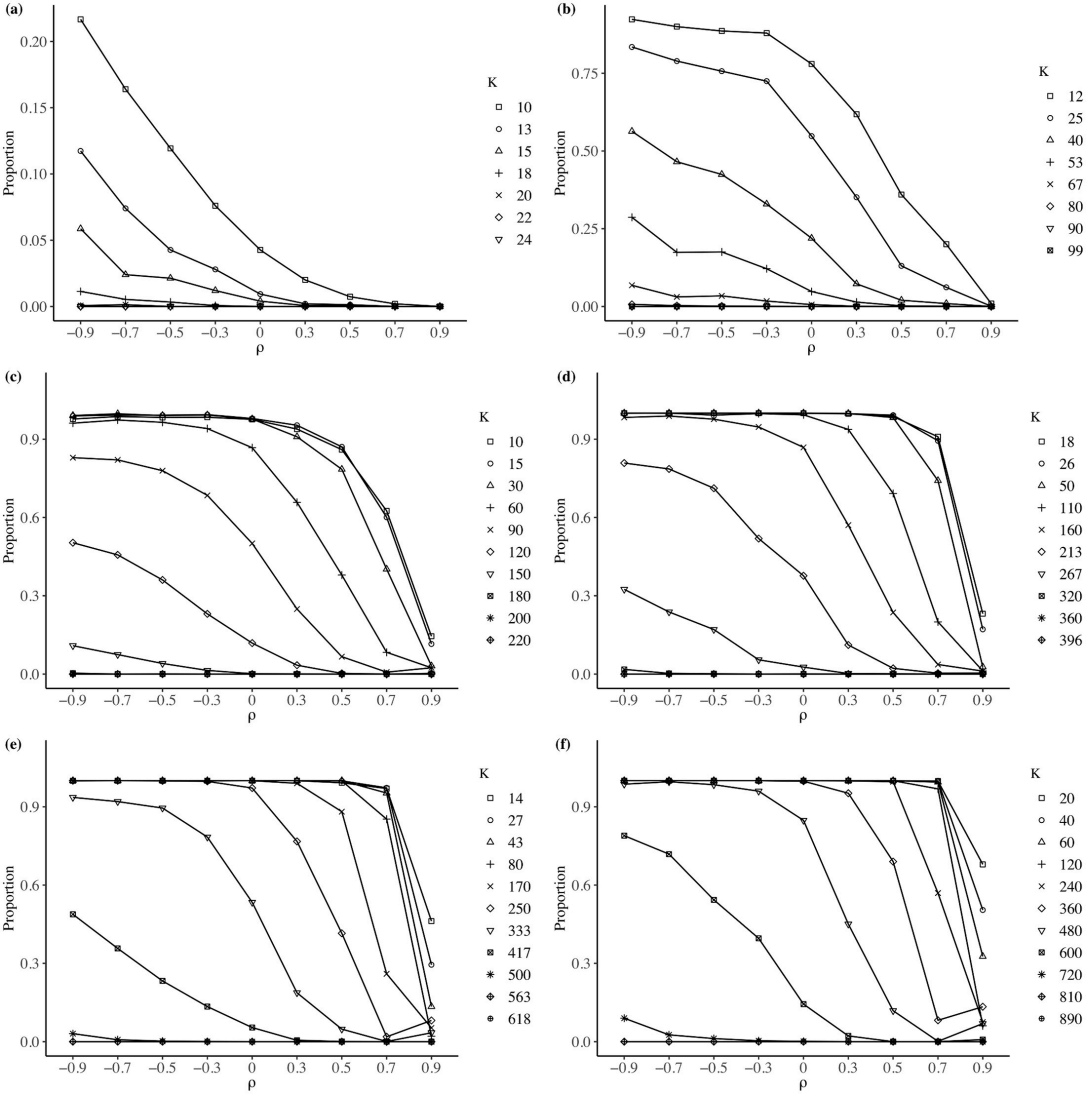
Proportion of instances for which the Levene test rejects the null hypothesis of equality of variance, with a level of significance *α* = 0.05. (a) *N* = 25; (b) *N* = 100; (c) *N* = 225; (d) *N* = 400; (e) *N* = 625; (f) *N* = 900.

To verify the MAUP effect on variance, we use the Levene test for equality between the variance of the original variable, $\sigma_{o}^{2}$, with the variance of each aggregated variable, $\sigma_{ag}^{2}$. Fig 5 shows the percentage of instances for which the Levene test rejects the null hypothesis $H_{0}:\;\sigma_{o}^{2}=\sigma_{ag}^{2}$, with *α* = 0.05. These results confirm that the MAUP effect decreases as either *k* or *ρ* increases.

**Fig 6:**
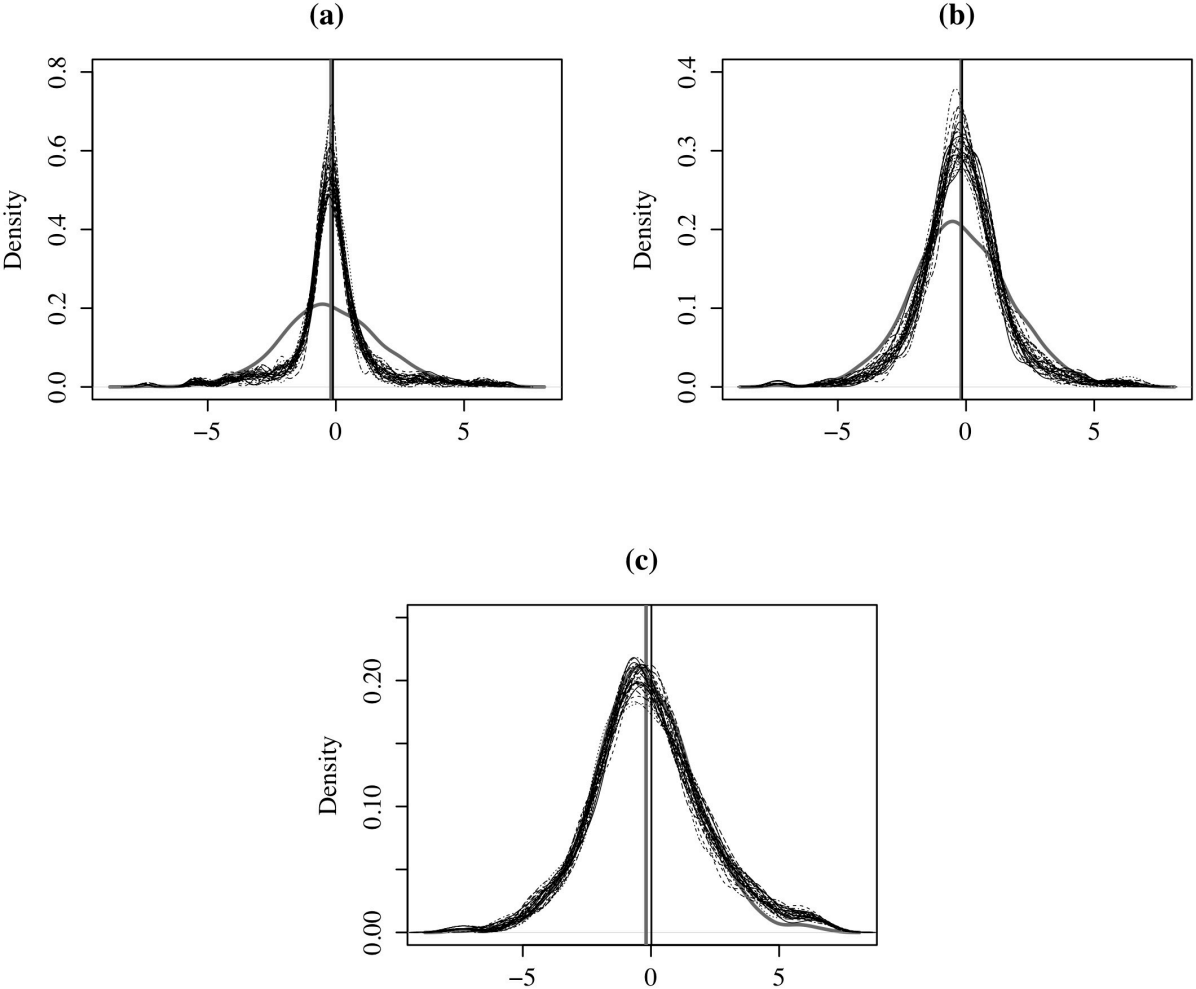
MAUP effects at three levels of spatial autocorrelation, (a) *ρ* = −0.9, (b) *ρ* = 0, and (c) *ρ* = 0.9. Solid line: original variable with *N* = 900; dashed lines: 30 aggregations with *k* = 240. The vertical lines indicate *μ*_*o*_ and *μ*_*ag*_.

Finally, in Fig 6 we present, for illustrative purposes, three instances with *ρ* = −0.9, *ρ* = 0.0 and *ρ* = 0.9 that aggregate *N* = 900 areas into *k* = 240 regions. These examples show how the MAUP fades as *ρ* increases.

## *S*-maup statistical test

Findings such as the effect of MAUP on variance and how MAUP fades as *ρ* and *k* increase are useful to find the functional form of our statistical test, *S*-maup, for measuring the level of sensitivity of a spatially distributed variable to the MAUP. We designed the test such that *S*-maup takes values close to zero when the variable is not sensitive to the MAUP and values close to one when the variable is highly sensitive to the MAUP. Furthermore, *S*-maup will be a univariate statistic applicable to spatially expansive variables whose aggregated values result from the average of the individual values.

### *S*-maup

**Fig 7:**
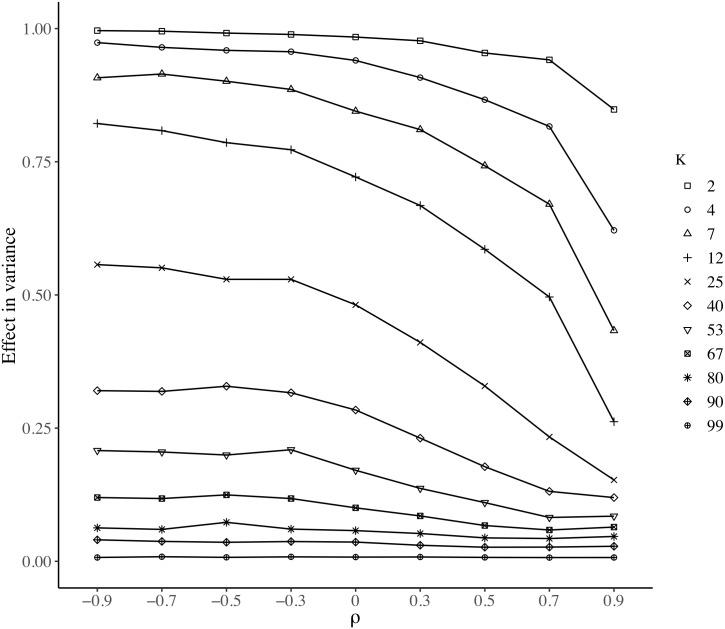
Median $\bar{RCV}$ for *N* = 100.

To find the functional form of *S*-maup, it is necessary design an expression that describes the distribution of the effects of MAUP on the variance ($\bar{RCV}$). To summarize those effects, we took the median of each Box Plot in Fig 4. Fig 7 shows an example of those summarized effects.

According to Fig 7, the mathematical expression of our test should take values close to one when the variable under evaluation has high negative spatial autocorrelation, *ρ* and is aggregated into a small number of regions, *k*. Conversely, the expression should take values close to zero when the variable under evaluation has high positive spatial autocorrelation, *ρ* and is aggregated into a large number of regions, *k*. Our expression should also be able to reproduce the way in which, for a given *k*, the MAUP effects decreases as *ρ* increases. Note that such a decrease is not the same for all values of *k*: when *k* is large, the effects of MAUP are low even for highly negative values of *ρ*; therefore, for a high *k*, the reduction of the MAUP effects, as *ρ* increases, are almost imperceptible. Thus, as *k* increases, our expression should modify the speed and moment at which the MAUP fades along *ρ*. Taking into account these different conditions, we started the construction of our *S*-maup statistic using an inverted logistic function [55], which is defined by Eq (5).

$$\begin{array}{c} M\left(\rho ;L,\eta ,\tau \right)=\frac{L}{1+\eta e^{\tau x}}\;\;,\; \end{array}$$ (5)

where *L* determines the maximum value of the curve; *η* determines the moment at which the curve begins to decline; and *τ* indicates the speed at which the curve declines. If we endogenize those three parameters, we should be able to approximate any line of the type shown in Fig 7. This is what we are going to develop in the rest of this subsection until we obtain an expression of *M* in which parameters *L*, *η* and *τ* depend on *ρ*, *k* and *N*.

**Fig 8:**
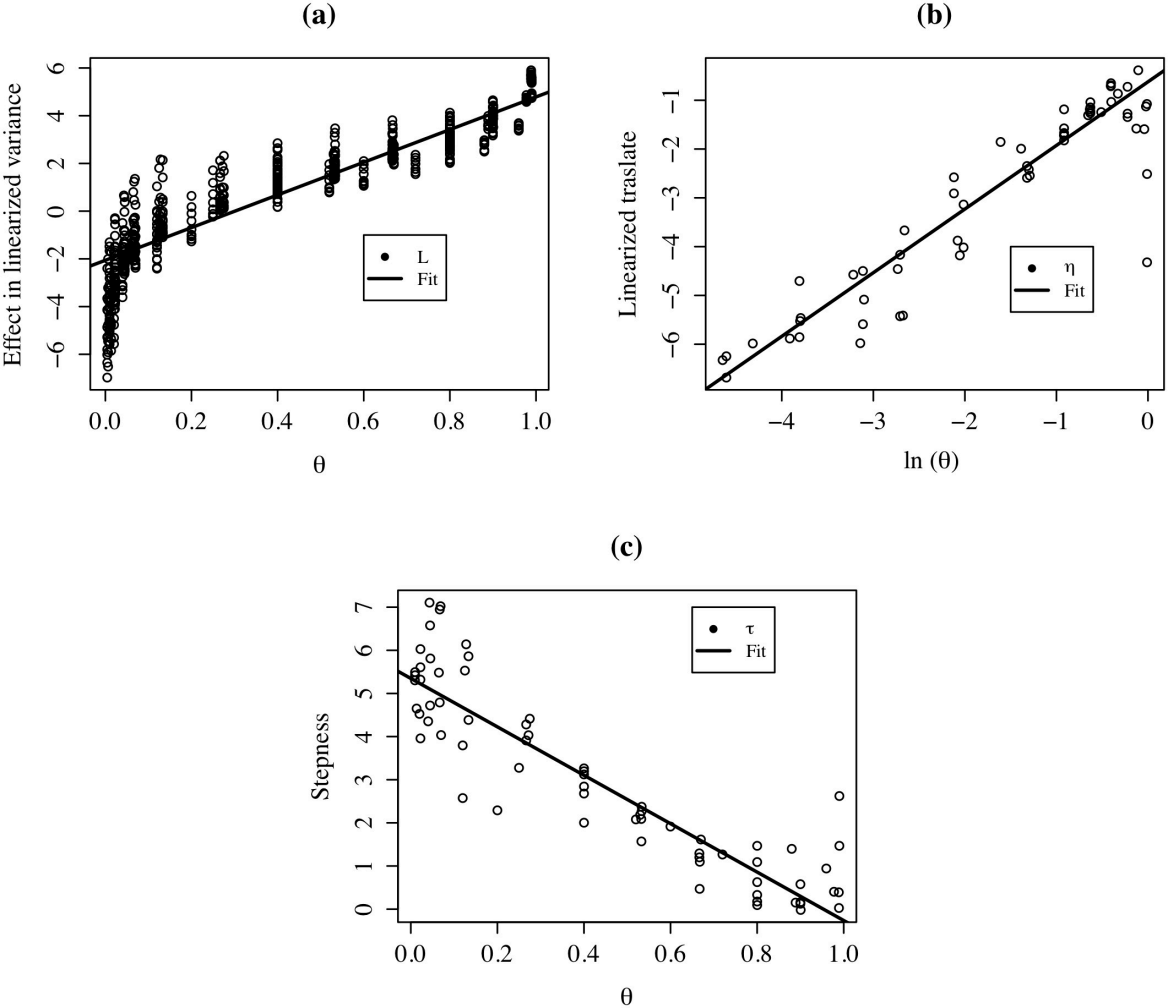
Adjustments of robust linear regression models. (a) Linearized logistic function (*L*); (b) Linearized power function (*η*); (c) Linear function (*τ*).

Starting with the parameter *L*, Fig 7 shows that the maximum value of each logistic curve depends on the level of aggregation *k*. This aggregation can be defined in relative terms as $\theta =\frac{k}{N}$. Therefore, the lower the level of aggregation (i.e., as *θ* approaches 1), the lower should be *L*. When plotting each median $\bar{RCV}$ against *θ*, it depicts an inverted “S” that could also be modeled as an inverse logistic function with the expression presented in Eq (6), whose linear form is given by Eq (7).

$$\begin{array}{c} L\left(\theta \right)=\frac{1}{1+e^{b+m\theta }}\;\;,\; \end{array}$$ (6)

$$\begin{array}{c} Ln(\frac{1-L}{L})=b+m\theta \;\;, \end{array}$$ (7)

where *b* and *m* are the parameters of the inverse logistic function. To estimate those parameters, we used a robust linear regression model that minimizes the influence of outliers. The parameter associated with *θ* is significant, and the adjusted R-squared = 86.7%. Fig 8(a) shows the robust regression over the linearized logistic function.

Returning to the logistic curves in Fig 7, both the moment at which the curves begin to decrease, *η*, and the speed of decreasing, *τ*, depend on *k*. Therefore, both parameters can be estimated as function of $\theta =\frac{k}{N}$. For this, we adjusted an inverse logistic function for each curve of the type presented in Fig 7. For each curve, the values of *η* and *τ* were calibrated using the optimized module of Scipy Python Library [56]. With this process, we obtained a value for *η* and *τ* for each value of *θ*. Then, we use a linearized power function, Eq (8), and a linear function, Eq (9), to express *η* and *τ* as a function of *θ*.

$$\begin{array}{c} \eta \left(\theta \right)=p\theta^{a}\;\; \end{array}$$ (8)

$$\begin{array}{c} \tau \left(\theta \right)=\beta_{0}+\beta_{1}\theta \;\; \end{array}$$ (9)

The parameter associated with *θ* was significant in both estimations and the adjusted *R*^2^, with 91.7% and 84.5% respectively. Fig 8b and 8c present the estimations.

Replacing the Eqs (6), (8) and (9) in (5) we have the Eq (10).

$$\begin{array}{c} M\left(\rho ,\theta \right)=\frac{\frac{1}{1+e^{b+m\theta }}}{1+p\theta^{a}e^{(\beta_{0}+\beta_{1}\theta )\rho }}\;\; \end{array}$$ (10)

The results of the estimation of the parameters in the robust linear regression model for the logistic function of *L* are as follows: *m* = 7.031 and *b* = −2.188. Considering that the model is estimated with the linearized logistic function, these results were transformed by natural logarithm. For the power function of *η*, the results are *p* = 0.516 and *a* = 1.287, because of the linearization of the power function, we applied the natural logarithm to the parameter *p*. Finally, the results of the linear function of *τ* are as follows: *β*_0_ = 5.319 and *β*_1_ = −5.532. Replacing in the equations produces the following:

$$\begin{array}{c} L\left(\theta \right)=\frac{1}{1+e^{-2.188+7.301\theta }}\;\; \end{array}$$ (11)

$$\begin{array}{c} \eta \left(\theta \right)=0.516\theta^{1.287}\;\; \end{array}$$ (12)

$$\begin{array}{c} \tau (\theta )=5.319-5.532\theta .\;\; \end{array}$$ (13)

Thus, the expression of the *S*-maup statistic is the following:

$$\begin{array}{c} M\left(\rho ,\theta \right)=\frac{\frac{1}{1+e^{-2.188+7.031\theta }}}{1+\left[0.516\theta^{1.287}\right]e^{[5.319-5.532\theta ]\rho }}\;\; \end{array}$$ (14)

Recall that *S*-maup statistic (*M*) is designed in such a way that for a bigger (smaller) sensitivity of a variable to the MAUP, the larger (smaller) is the value of *M*. This characteristic allows us to define a non-parametric unilateral statistical test, which is stated below:

*H*_0_: *The variable y*_*i*_ *is not significantly affected by the MAUP.*
*H*_1_: *The variable y*_*i*_ *is significantly affected by the MAUP.*

Where the statistic for the test is given by Eq (14), and therefore, *H*_0_ will be rejected if the statistic value belongs to the rejection region (*RR*) defined in Eq (15).

$$\begin{array}{c} RR=\{M|M>M_{\alpha ;\rho ,N}\}\;\; \end{array}$$ (15)

*M*_*α*;*ρ*,*N*_ is the critical value given a significance level *α*, a level of spatial autocorrelation (*ρ*), and a number of areas (*N*). We implemented a Monte Carlo simulation to find the empirical distribution of the *S*-maup under the null hypothesis previously stated. The empirical distribution allows us to obtain the critical values as well as the pseudo-value *p* to determine the proof significance.

### Critical values and *p*-value

To calculate the critical values, we performed an exhaustive simulation study based on non-parametric statistic methodology. Recall that *H*_0_ means no sensitivity of a variable to MAUP, which is equivalent to stating that, for a given *k*, the variance of the aggregated variable is statistically equal to the variance of the original variable. For building the empirical distribution under *H*_0_, we set a value for *N* and *ρ* and generated an SAR process with parameters (*N*, *ρ*). Then, we randomly selected an integer value *k* such that 0.1*N* < *k* < *N*, thus yielding 30 random aggregations of the variable into *k* regions. Next, we applied the Levene test for equality of variances between the original variable and each one of the 30 aggregated variables. The SAR(*N*, *ρ*) variable was kept if and only if the Levene test was not rejected in all 30 cases. If there was at least one rejection, then we chose, at random, a new *k* and repeated the previous steps. This procedure was repeated until we obtained 1,000 instances for each pair (*N*, *ρ*). We then calculated the *S*-maup statistic for those instances using Eq (14) and generated the empirical distribution of the statistics under *H*_0_. The critical values were obtained by calculating the 90%, 95%, 99% percentiles for the empirical distribution. Table 3 presents the table of critical values. This Table implied the generation of 54,000 instances.

**Table 3:** Critical values (*M*_*α*;*ρ*,*N*_).

	Number of areas (*N*)						
*ρ*	*α*	25	100	225	400	625	900
-0.9	0.01	0.83702	0.09218	0.23808	0.05488	0.07218	0.02621
	0.05	0.83699	0.08023	0.10962	0.04894	0.04641	0.02423
	0.1	0.69331	0.06545	0.07858	0.04015	0.03374	0.02187
-0.7	0.01	0.83676	0.16134	0.13402	0.06737	0.05486	0.02858
	0.05	0.83662	0.12492	0.08643	0.05900	0.04280	0.02459
	0.1	0.79421	0.09566	0.06777	0.05058	0.03392	0.02272
-0.5	0.01	0.83597	0.16524	0.13446	0.06616	0.06247	0.02851
	0.05	0.83578	0.13796	0.08679	0.05927	0.04260	0.02658
	0.1	0.68900	0.10707	0.07039	0.05151	0.03609	0.02411
-0.3	0.01	0.83316	0.19276	0.13396	0.06330	0.06090	0.03696
	0.05	0.78849	0.16932	0.08775	0.05464	0.04787	0.03042
	0.1	0.73592	0.14282	0.07076	0.04649	0.04001	0.02614
0.0	0.01	0.82370	0.17925	0.15514	0.07732	0.07988	0.09301
	0.05	0.81952	0.15746	0.11126	0.06961	0.06066	0.05234
	0.1	0.71632	0.13621	0.08801	0.06112	0.04937	0.03759
0.3	0.01	0.76472	0.23404	0.24640	0.11588	0.10715	0.07070
	0.05	0.70466	0.21088	0.15360	0.09766	0.07938	0.06461
	0.1	0.63718	0.18239	0.12101	0.08324	0.06347	0.05549
0.5	0.01	0.67337	0.28921	0.25535	0.13992	0.12975	0.09856
	0.05	0.59461	0.23497	0.18244	0.11682	0.10129	0.08860
	0.1	0.46548	0.17541	0.14248	0.10008	0.08137	0.07701
0.7	0.01	0.52155	0.47399	0.29351	0.23923	0.20321	0.16250
	0.05	0.48958	0.37226	0.22280	0.20540	0.16144	0.14123
	0.1	0.34720	0.28774	0.18170	0.16442	0.13395	0.12354
0.9	0.01	0.28599	0.28938	0.43520	0.44060	0.34437	0.55967
	0.05	0.21580	0.22532	0.27122	0.29043	0.23648	0.31424
	0.1	0.17640	0.18835	0.21695	0.23031	0.19435	0.22411

Following the percentile approach utilized by [57], we can calculate a pseudo-*p*-value for a given value of the *S*-maup test (*M*), using the Eq (16):

$$\begin{array}{c} P\left(M\right)=\frac{1}{1,000}\sum_{j=1}^{1,000}\Psi \;\;,\; \end{array}$$ (16)

where Ψ = 1 if $M_{j}^{\rho ,N}>M$, Ψ = 0 otherwise. The vector $M_{j}^{\rho ,N}$ comes from the simulations performed to produce Table 3. Since those vectors are extremely computationally intensive to produce (in some instances requiring months of supercomputer computation for completion), they will be publicly available at http://www.___.edu, as well as the Python script to run the *S*-maup statistic.

Table 4 presents some examples of the *S*-maup statistic for different values of *N* and *k*. Note that when the variable *y*_*i*_ presents characteristics against the null hypothesis (*H*_0_), then the *M* value of the *S*-maup should be greater than the critical value at some significance level *α*, and therefore, the pseudo-value *p* of the test must be smaller than the significance level. If *H*_0_ is rejected, it can be concluded that the variable *y*_*i*_ is sensitive to the MAUP, and therefore, a MAUP effect exists when aggregating *y*_*i*_ in *k* regions.

**Table 4:** Example *S*-maup.

*Variable*	*N*	*k*	*ρ*	*M*	*M*_*α*;*ρ*,*n*_	Pseudo-v *p*
$y_{i}^{1}$	1,000	400	0.007	0.24002	0.05234	0.0 ***
$y_{i}^{2}$	1,000	600	0.007	0.05871	0.05234	0.034 **
$y_{i}^{3}$	1,000	800	0.007	0.01187	0.05234	0.616
$y_{i}^{4}$	500	100	-0.634	0.09237	0.05900	0.0 ***
$y_{i}^{5}$	500	280	-0.634	0.05466	0.05900	0.078 *
$y_{i}^{6}$	500	380	-0.634	0.00767	0.05900	0.852
$y_{i}^{7}$	220	60	0.562	0.32197	0.18244	0.00 ***
$y_{i}^{8}$	220	90	0.562	0.18513	0.18244	0.046 **
$y_{i}^{9}$	220	150	0.562	0.04357	0.18244	0.443
$y_{i}^{10}$	150	15	0.801	0.29201	0.22532	0.009 **
$y_{i}^{11}$	150	50	0.801	0.08072	0.22532	0.366
$y_{i}^{12}$	150	90	0.801	0.00997	0.22532	0.883

*** *p* < 0.01,

** *p* < 0.05,

* *p* < 0.1.

Note that when the spatial autocorrelation is highly positive (e.g., *ρ* = 0.801), the variable allows high levels of aggregation. The results also confirm that low levels of spatial aggregation do not lead to the undesirable effects of MAUP.

## Power and size

The power is a natural way of evaluating the test performance. It is defined as the probability of rejecting the null hypothesis, given that the null hypothesis is false. In other words, it is the probability of not committing a type II error (*β*); thus, the power is (1 − *β*). In our context, the power means the probability that sufficient statistical evidence exists in the sample to affirm that the variable *y*_*i*_ is affected by the MAUP, when in fact, the variable *y*_*i*_ is affected by the dimensions of the MAUP. Hence, it is expected that the power of the test is close, or equal, to 1.

Since *H*_1_ implies that the variance of the original variable is different from the variance of the aggregate variable, we implemented the following simulation experiment to measure the power of our statistical test: For each tuple (*N*, *ρ*), with *N* ∈ {100, 400, 900} and *ρ* ∈ {±0.9, ±0.7, ±0.5, ±0.3,0}. Given a tuple (*N*, *ρ*) we generate an SAR process and perform 30 random spatial aggregations of the *N* areas into *k* regions such that *k* is selected at random as an integer value such that 0.1*N* < *k* < *N*. The SAR process is kept if and only if the Leven test between the original variable and each one of the 30 aggregated variables is rejected. We repeat this process until we generate 1,000 valid instances for each tuple (*N*, *ρ*). Each entry in Table 5 reports the proportion of 1,000 instances for which our test rejects *H*_0_. Because most values are close to 1, we can argue that our *S*-maup is highly effective in identifying variables that are sensitive to the MAUP effect.

**Table 5:** Estimated power of *S*-maup.

*ρ*	Number of areas (*N*)		
	*N* = 100	*N* = 400	*N* = 900
-0.9	0.989	0.985	0.997
-0.7	0.986	0.996	1.000
-0.5	0.981	0.998	1.000
-0.3	0.982	0.998	1.000
0.0	0.997	0.999	0.999
0.3	0.986	0.996	1.000
0.5	0.986	0.996	0.999
0.7	0.783	0.985	0.995
0.9	0.977	0.703	0.492

Level of significance *α* = 0.05.

Test size is also a way of evaluating the test performance. Test size is defined as the probability of rejecting the null hypothesis given that the null hypothesis is true. In other words, it is the probability of committing a type I error (*α*). In our context, test size means the probability that sufficient statistical evidence exists in the sample to affirm that the variable *y*_*i*_ is affected by the MAUP, when in fact the variable *y*_*i*_ is not. Hence, it is expected that the proportion of instances for which our test commits type I error is close the theoretical significance level (*α*).

The empirical test size is calculated following a similar procedure implemented to calculate the power, but in this case, the tuple (*N*, *ρ*) is selected if and only if the Levene test is not rejected in all 30 cases. Table 6 reports the size of our test, which show the best performance in scenarios of positive spatial autocorrelation.

**Table 6:** Estimated size of *S*-maup.

*ρ*	Number of areas (*N*)		
	*N* = 100	*N* = 400	*N* = 900
-0.9	0.163	0.087	0.065
-0.7	0.080	0.037	0.080
-0.5	0.091	0.043	0.083
-0.3	0.073	0.097	0.136
0	0.102	0.066	0.026
0.3	0.081	0.057	0.038
0.5	0.098	0.062	0.032
0.7	0.043	0.032	0.045
0.9	0.110	0.024	0.009

Level of significance *α* = 0.05.

## An illustrative application of the *S*-maup test

In this section, we present an empirical illustration within the context of a Mincer wage equation [58] that explains the salary based on schooling and experience. Eq (17) presents the most basic version of the Mincer wage equation.

$$\begin{array}{c} LNW=\beta_{0}+\beta_{1}*YRSCHOOL+\beta_{2}*EXP+\beta_{3}*EXP^{2}+\varepsilon ,\; \end{array}$$ (17)

where *LNW* is the natural logarithm of income (hourly wage), *YRSCHOOL* years of schooling, *EXP* years of potential labor market experience (calculated as the age in years minus years of education plus 6), and *ε* is a mean zero residual. It is important to clarify that this example is merely illustrative. We use this equation because its simplicity allows us to present a simple application of our test.

We use the 2011 census data from South Africa retrieved from the Integrated Public Use Microdata Series, International (IPUMS-International), at the Minnesota Population Center [59]. The data include 688,310 individuals who were working at the time of the survey. We aggregate the individual data into 206 municipalities using the weighted average of individual incomes, years of schooling, and the potential work experience.

**Fig 9:**
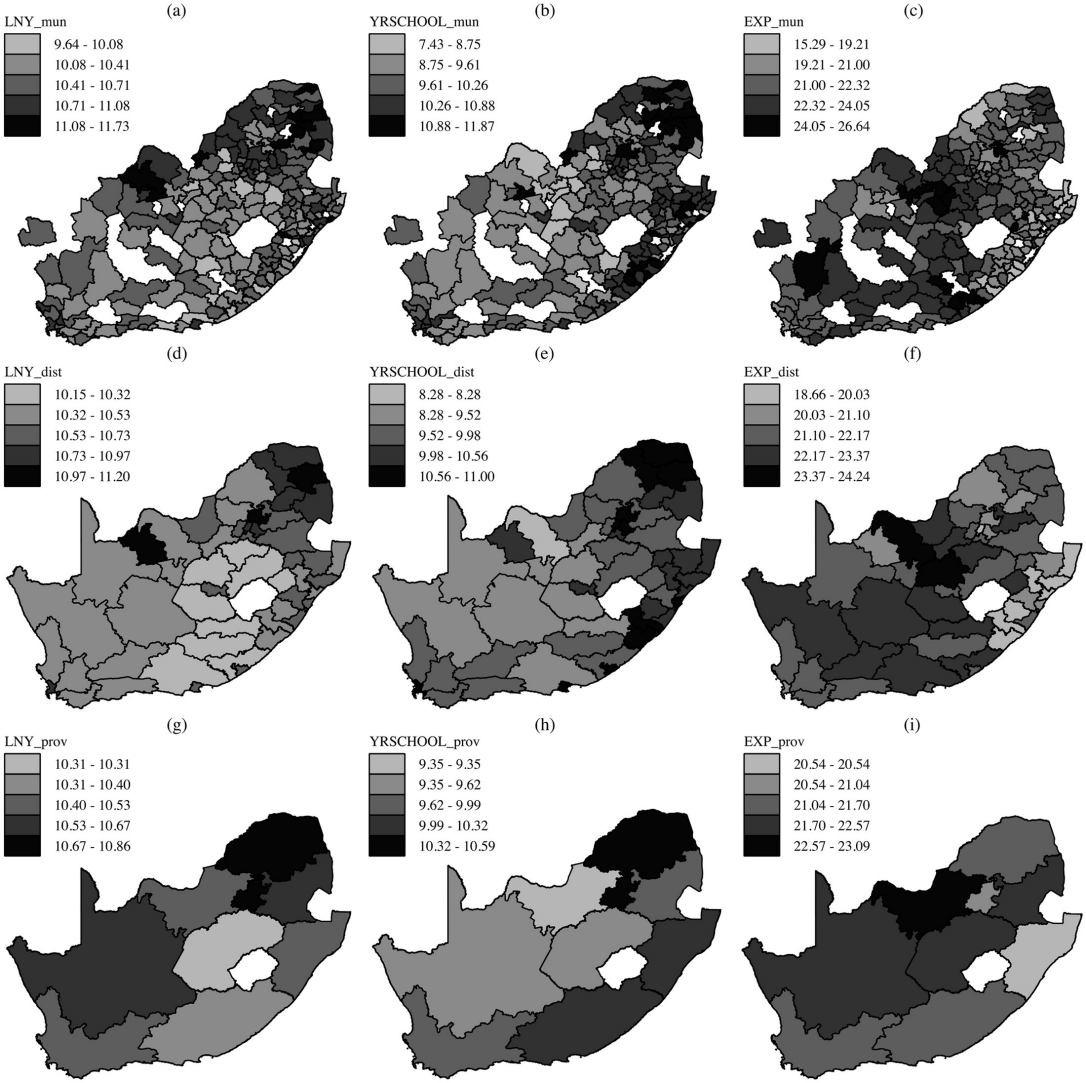
Municipalities: (a), (b) and (c). Districts: (d), (e) and (f). Provinces: (g), (h) and (i).

The 206 municipalities are our basic unit of analysis (i.e., our disaggregated variable). Other administrative units in South Africa include 52 districts and 9 provinces. Table 7 shows some descriptive statistics of our variables at the three administrative levels. Note how the standard deviation of the three variables narrows as the level of aggregation increases. The spatial distribution of the variables is presented in Fig 9.

**Table 7:** Descriptive statistics.

Variable	Municipalities				
	Obs.	Mean	Desv. Std.	Mín.	Máx.
LNW	206	10.51	0.35	9.64	11.73
YRSCHOOL	206	9.95	0.81	7.43	11.87
EXP	206	21.69	1.71	15.28	26.64
	Districts				
LNW	52	10.56	0.25	10.15	11.20
YRSCHOOL	52	10.06	0.61	8.28	10.99
EXP	52	21.59	1.21	18.66	24.24
	Provinces				
LNW	9	10.57	0.19	10.31	10.86
YRSCHOOL	9	10.00	0.44	9.35	10.59
EXP	9	21.77	0.77	20.54	23.09

Table 8 presents the estimation at the municipal level. The coefficients of education and experience are significant and exhibit the expected signs.

**Table 8:** Mincer model estimate: South Africa.

LNW	Coef.	Desv. Std	*p* > |*t*|	Confidence Interval at 95%	
YRSCHOOL	0.3364	0.0259	0.000 ***	0.2852	0.3876
EXP	0.4008	0.1499	0.008 ***	0.1051	0.6965
EXP2	-0.0085	0.0034	0.016 **	-0.0153	-0.0016
CONST.	2.4796	1.6243	0.128	-0.7232	5.6825
Num. Obs. 206					
F(3,202) = 68.84					
*R*^2^ adjusted = 0.498					

*** *p* < 0.01,

** *p* < 0.05,

*p < 0.1.

What would be the maximum level of spatial aggregation for which these results hold? Note that here we are asking about the minimum value for *k* that preserves the distributional characteristics of the variables; we are not aiming to evaluate a specific regionalization for a given value of *k*. We can use our *S*-maup statistic to answer this question by identifying the minimum value of *k* for which our test fails to reject the null hypothesis of no influence of the MAUP. In Table 9, we present the results of our test for different levels of spatial aggregations. For this, our test requires the level of spatial autocorrelation of each variable (*ρ*) and the value of $\theta =\frac{k}{N}$. Note that at *k* = 135, the *S*-maup indicates that the variable *LNW* is affected by the MAUP. This finding may imply that the results obtained at municipal level (*k* = 206) may hold until an aggregation level of *k* = 136 that is the aggregation level at which all the variables involved in the regression do not lose their distributional characteristics. Another conclusion from these results is that the results obtained at the municipal level do not hold at district or province levels.

**Table 9:** Estimator of the statistic *S* -maup: South Africa.

	*N* = 206							
	LNW		YRSCHOOL		EXP		EXP2	
	*ρ* = 0.05		*ρ* = 0.25		*ρ* = 0.24		*ρ* = 0.40	
*k*	*M*	Ps-v *p*	*M*	Ps-v *p*	*M*	Ps-v *p*	*M*	Ps-v *p*
200	0.011	0.806	0.011	0.820	0.011	0.819	0.011	0.833
180	0.022	0.589	0.021	0.619	0.021	0.619	0.020	0.656
150	0.057	0.242	0.052	0.330	0.053	0.327	0.048	0.414
136	0.087	0.101	0.079	0.197	0.079	0.194	0.072	0.302
135	0.091	0.094 *	0.081	0.187	0.082	0.185	0.073	0.295
134	0.093	0.089 *	0.083	0.181	0.084	0.179	0.076	0.290
132	0.099	0.077 *	0.088	0.166	0.089	0.166	0.079	0.273
124	0.124	0.036 **	0.111	0.115	0.112	0.114	0.099	0.208
122	0.131	0.032 **	0.117	0.107	0.118	0.104	0.104	0.186
120	0.139	0.025 **	0.123	0.094 *	0.125	0.091 *	0.110	0.167
118	0.147	0.019 **	0.131	0.081 *	0.132	0.080 *	0.142	0.101
110	0.182	0.003 **	0.161	0.043 **	0.163	0.042 **	0.149	0.093 *
108	0.192	0.001 **	0.169	0.034 **	0.172	0.033 **	0.149	0.093 *
52	0.584	0.000 ***	0.527	0.001 ***	0.533	0.001 ***	0.461	0.00 ***
9	0.863	0.000 ***	0.847	0.001 ***	0.849	0.001 ***	0.822	0.00 ***

*** *p* < 0.01,

** *p* < 0.05,

*p < 0.1.

**Fig 10:**
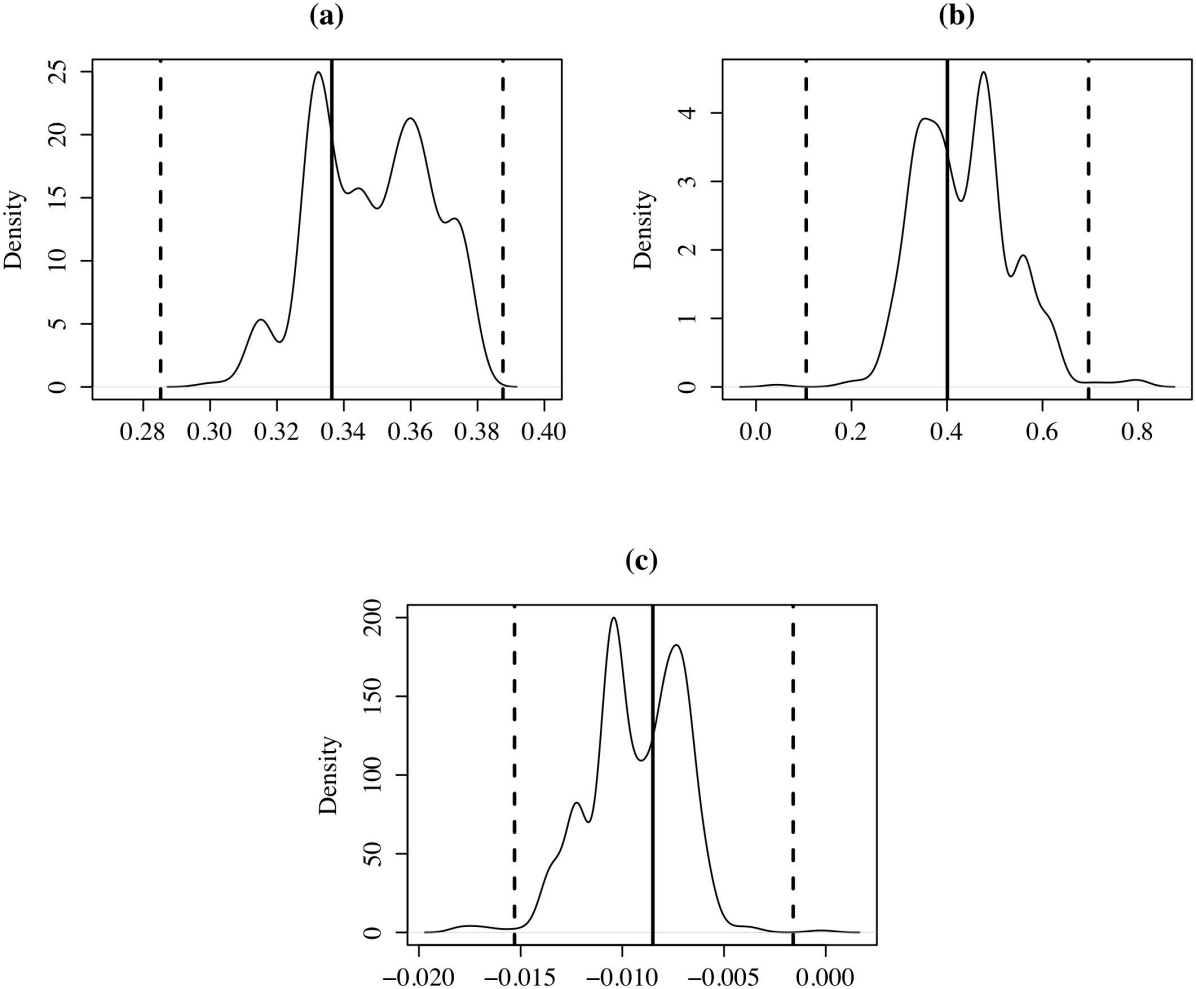
Distribution of coefficients, *k* = 136: (a) YRSCHOOL; (b) EXP; (c) EXP2. Horizontal black line: coefficient (206 municipalities), dashed lines are the respective confidence intervals 95%.

Fig 10 compares the coefficients obtained at the municipal level (black and dashed vertical lines) with the distribution of the coefficients obtained by estimating the Mincer equation on 1,000 random spatial aggregations of the *k* = 206 municipalities into *k* = 136 regions. Fig 10a, corresponding to years of education, shows that 100% of the coefficients estimated with *k* = 136 fall into the 95% confidence intervals. Fig 10b, corresponding to years of experience, shows that 98.8% of the coefficients estimated with *k* = 136 fall into the 95% confidence intervals. Finally, Fig 10c, corresponding to the squared years of experience, shows that 98.7% of the coefficients estimated with *k* = 136 fall into the 95% confidence intervals.

**Fig 11:**
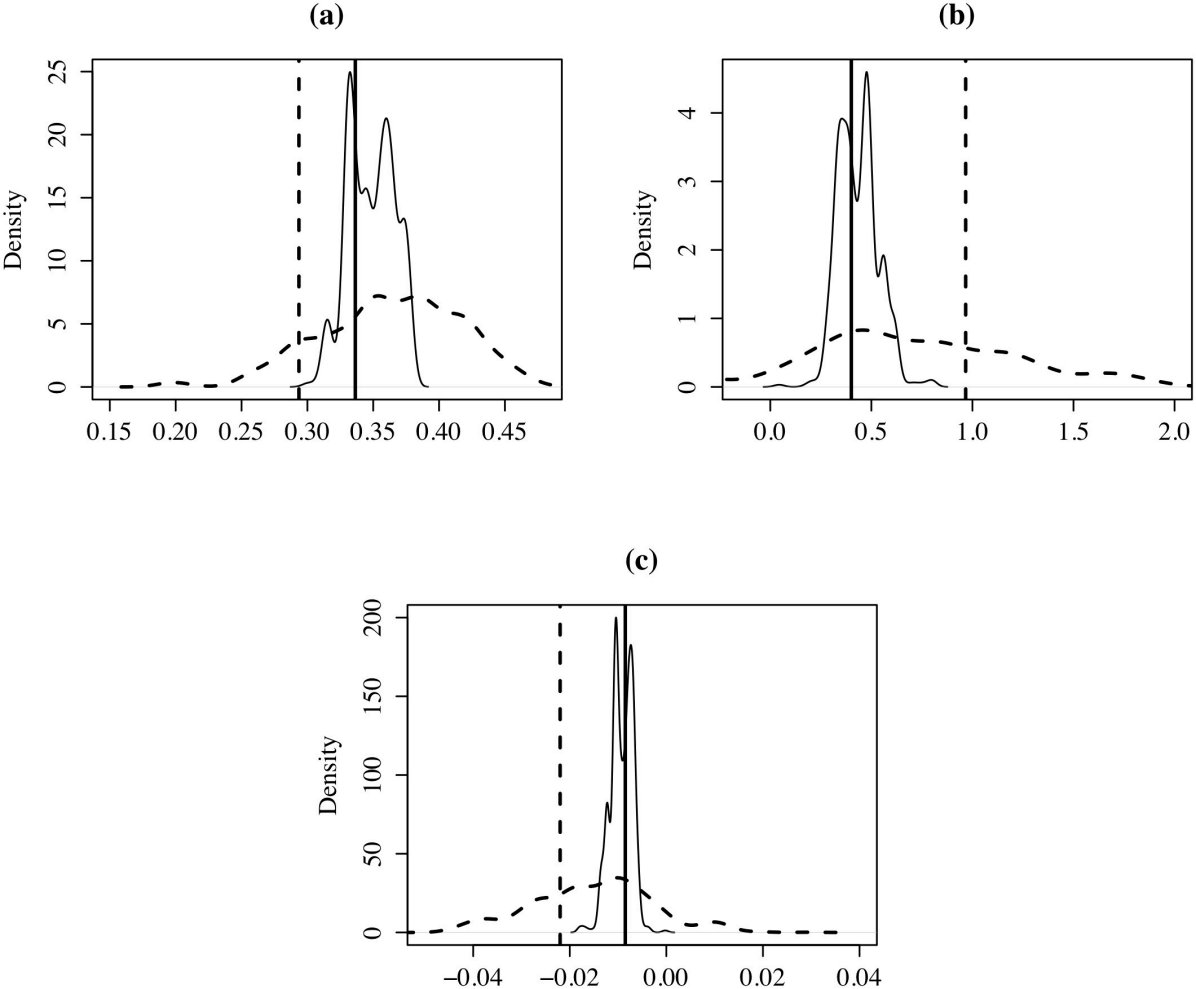
Distribution of coefficients. Line:*k* = 136, dotted line:*k* = 52: (a) YRSCHOOL; (b) EXP; (c) EXP2. horizontal black line: coefficient (206 municipalities). horizontal dotted line: coefficient (52 districts).

Next, we estimated the Mincer model for *k* = 52 and compared it with the estimation for *k* = 206 municipalities. As we did previously, we made 1,000 random aggregations and obtained the distribution of the estimated coefficients for *K* = 136 and *k* = 52. Fig 11 shows how the estimations with *k* = 52 are more volatile and deviated than those with *k* = 136 regions. Note also that the coefficients for *K* = 206.

## Conclusions

This paper introduced the first statistic of its kind for measuring the level of sensitivity of a spatially expansive variable to the MAUP. The statistic requires as input parameters the level of aggregation $\theta =\frac{k}{N}$ and the level of spatial autocorrelation of the variable *ρ*. The results indicate that, in general, both the statistical size and power improve with increasing sample size. We also provide the table of critical values and a procedure to calculate the pseudo-*p* value of the test.

The empirical application shows the usefulness of the test for identifying the maximum level of aggregation at which the original variable preserves its distributional characteristics. Additionally, it can be useful to test whether two aggregation levels are comparable.

We recognize that the main properties of the *S*-maup were obtained from an empirical simulation procedure, and they rely more heavily on hard experimental computation than theoretical methods. However, the complexity of the question addressed in this paper may explain why this is the first attempt to answer it even though the MAUP has been in the literature since the late 1970s. We hope that this first attempt motivates other researchers to contribute other approaches to answer the same question.

Further research could focus on the formulation of a *S*-maup test that includes the average number of neighbors as an additional parameter and makes a robust version of the statistic that allows for any specification of the weights matrix. Also, the formulation of a variogram-based *S*-maup version could be of great interest for researchers in the field of geostatistics (we acknowledge one of the anonymous referees for this suggestion).
